# A single, episodic event of unilateral/bilateral scrotal swelling in a group of adult boars at an Austrian boar stud

**DOI:** 10.1186/s40813-023-00313-1

**Published:** 2023-07-14

**Authors:** Lukas Schwarz, Sophie Dürlinger, Vera Martin, Herbert Weißenböck, Rene Brunthaler, Till Rümenapf, Angelika Auer, Igor Loncaric, Irene Zimpernik, Nicole Reisinger, Bettina Behler-Wöchtl, Dragos Scarlet, Gary Althouse, Chris Kuster, Johannes Kauffold, Joaquim Segales, Martine Laitat, Pierre Thilmant, Alexander Grahofer, Andrea Ladinig

**Affiliations:** 1 Department for Farm Animals and Veterinary Public Health, University Clinic for Swine, University of Veterinary Medicine Vienna, Vienna, Austria; 2 Department of Pathobiology, Institute of Pathology, University of Veterinary Medicine Vienna, Vienna, Austria; 3 Department of Pathobiology, Institute of Virology, University of Veterinary Medicine Vienna, Vienna, Austria; 4 Department of Pathobiology, Institute of Microbiology, University of Veterinary Medicine Vienna, Vienna, Austria; 5 Austrian Agency for Health and Food Safety, Institute for Veterinary Disease Control, Mödling, Austria; 6 DSM Austria GmbH, Getzersdorf, Austria; 7 DSM, BIOMIN Research Center, Tulln an der Donau, Austria; 8 Obstetrics, Gynaecology and Andrology, Department for Companion Animals and Horses, University of Veterinary Medicine Vienna, Vienna, Austria; 9 Department of Clinical Studies-New Bolton Center, School of Veterinary Medicine, University of Pennsylvania, Philadelphia, USA; 10 Kuster Research and Consulting, Inc., Atkinson, USA; 11 Unitat Mixta d’Investigació IRTA-UAB en Sanitat Animal, Centre de Recerca en Sanitat Animal (CReSA), and Departament de Sanitat i Anatomia Animals, Facultat de Veterinària, Campus de la Universitat Autònoma de Barcelona (UAB), 08193 Bellaterra, Catalonia Spain; 12 Clinic for Swine, Faculty of Veterinary Medicine, University of Liège, Liège, Belgium; 13 Centre d’Insémination Artificielle Porcin (CIAP), Argenteau, Province de Liège Belgium; 14 Clinic for Swine, Department for Clinical Veterinary Medicine, Vetsuisse Faculty, University of Bern, Bern, Switzerland; 15 University of Leipzig, Leipzig, Germany; 16 Present Address: Clinic of Reproductive Medicine and Institute for Veterinary Anatomy, Vetsuisse Faculty, University of Zurich, Zurich, Switzerland

**Keywords:** Scrotal enlargement, Orchitis, Scrotal swelling, Swine, Boar

## Abstract

**Background:**

Scrotal swelling is a clinical situation which can be caused by different aetiologies. In this case report, we describe a multi-week episode of unilateral and bilateral scrotal swelling in boars at an Austrian boar stud and its diagnostic work-up.

**Case presentation:**

In the summer of 2020, the herd veterinarian of an Austrian boar stud reported that over a period of six weeks, five out of 70 boars presented with unilateral severe swelling of the left scrotum and three out of 70 boars with bilateral severe swelling of the left and moderate swelling of the right scrotum, respectively. A complete history was obtained and an on-site evaluation of the facility was done. Five boars were necropsied, and a variety of samples harvested for further diagnostic investigations. Infectious differential diagnoses associated with unilateral swelling of the scrotum or the testis were excluded through serological and tissue testing. In three of the five boars, histopathology revealed complete acute haemorrhagic necrosis of the left testis concurrent with strongly congested blood vessels. Review of the collected information with a group of experts in the field of boar stud management resulted with consensus that, most likely, trauma was the etiologic event causing the clinical signs and pathology. Coincident with discussion of implementing video recording cameras in the boar housing area, no further clinical cases followed. As this case occurred during the first lockdown of the COVID-19 pandemic, we propose that the distress and travelling restrictions may have contributed to frustration among boar stud workers, which was consequently expressed as misbehaviour against boars.

**Conclusions:**

Once all known infectious causes of unilateral swelling of the scrotum were excluded, a critical diagnostic work-up focused on non-infectious causes. Non-infectious causes, such as trauma, need to be carefully evaluated, as it may also include human misbehaviour against boars. Summarizing all findings of this case report, the authors hypothesize that a blunt trauma was the reason for the series of mainly unilateral swelling of the scrota of boars.

**Supplementary Information:**

The online version contains supplementary material available at 10.1186/s40813-023-00313-1.

## Background

Boar studs are closed husbandry systems designed for semen production for artificial insemination of breeding sows. Management, veterinary support and animal care are generally done following higher standards compared to conventional piglet producing farms, with all procedures in boar studs being well structured and defined. This is an essential aspect because the health status of boars used for semen production is reflected in the quality and quantity of their semen [1]. Regular monitoring and surveillance of diseases and of semen quality aids in quickly detecting abnormalities in a boars’ general health status [2]. In Austria, health monitoring in boar studs consists of routine serological profiling for detection of antibodies against infectious disease pathogens (in other words, classical swine fever virus, suid herpesvirus 1, porcine reproductive and respiratory syndrome virus (PRRSV), *Brucella* spp., *Leptospira* spp.), PCR-based investigations regarding PRRSV, and daily observation of boar’s general behaviour and willingness to mount a phantom for semen collection. Apathy, anorexia or unwillingness to mount are strong indicators of sickness due to various etiologic factors.

The establishment of quality assurance programmes for boar health is essential for production of high-quality semen [3]. This implicates that in case of abnormalities, either in boars or in collected semen/ejaculates, actions are required by the herd veterinarian(s) to re-establish the quality standards in a boar stud. Primary reasons for culling boars at stud include reproductive problems (for example, boar subfertility/infertility, low libido, poor semen quality, and genitalia problems) that represents 23.7–26.4% of all cullings, followed by lameness/leg problems (8.4–14.9%), and age (9.3–28.5%) [4–6]. Isolated cases of boars with abnormalities may not be alarming in regards of the whole herd, but if several cases with similar clinical presentation occur over a short time period, this is a sign that veterinary intervention is urgently required. However, every single case needs urgent veterinary intervention as such isolated cases may be painful events.

In this case report we describe a multi-week episode of unilateral and bilateral scrotal swelling in several boars at an Austrian boar stud.

## Case presentation

### Anamnesis

**Fig. 1:**
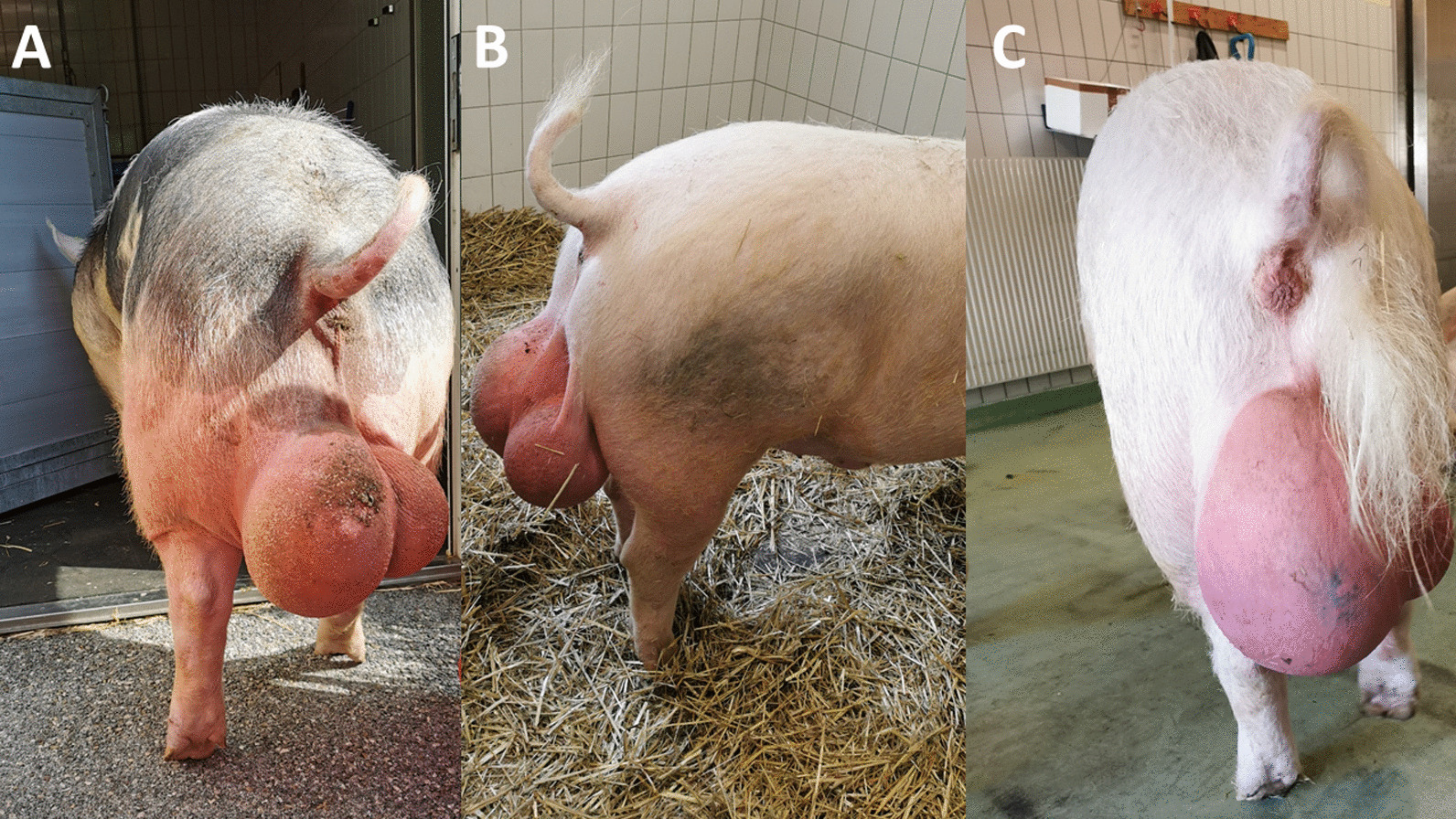
Selected boars with severely swollen left scrota. **A** Piétrain, boar 1 **B** Large White, boar 2 **C** Large White, boar 5

In the summer of 2020, the herd veterinarian of an Austrian boar stud observed an abrupt onset of primarily unilateral swelling of the left part of scrota and testis of boars (~ 11.43%; 8/70 boars; Fig. 1) which extended over a period of six weeks. Animal caretakers reported that the swelling of the scrota and testes developed over approximately one month until it reached its final size. Initially, the veterinarian suspected a recently introduced infectious disease that was affecting the boars showing clinical signs. New boars were kept in a locally separated quarantine unit for a minimum of six weeks before they were introduced into the resident herd. During quarantine and thereafter each boar of that stud got tested serologically on a regular basis for notifiable diseases (in other words, African and classical swine fever, Aujeszky’s disease, brucellosis) and for other diseases relevant to pig reproduction (for example, PRRSV, leptospirosis, chlamydiosis). All boars were regularly vaccinated against *Erysipelothrix rhusiopathiae* and porcine parvovirus. Out of the 70 boars kept in the resident stud, eight boars of different breeds (Piétrain, Duroc, German Landrace and Large White) and ages (one to seven years of age) were affected. Five boars showed unilateral swelling of the scrotum, and three boars presented with bilateral swelling with a more pronounced swelling on the left side as the main clinical sign, respectively, without expression of pain. Six out of the eight affected boars were held in pens located in the same row at the stud. Initially, two boars (two- and three-year-old Large White and Piétrain animals respectively) were forwarded by the herd veterinarian to the University Clinic for Swine, University of Veterinary Medicine Vienna, Vienna, Austria for further investigation and a diagnostic work up. During a subsequent on-site visit to the boar stud by the University Clinic for Swine, three additional boars with swollen scrota were observed and selected for a detailed diagnostic work up at the University of Veterinary Medicine Vienna, Vienna, Austria. Furthermore, ejaculates from six of the affected boars showing various severity of scrotal swelling and from two boars with no obvious signs of scrotal swelling as controls were collected for evaluation of sperm morphology. The “control” boars were kept in the same row, but never showed any signs of enlargement of the scrotum nor any other clinical signs and therefore were selected as healthy controls.

### Clinical examination

Clinically, all five examined boars showed mild (n = 1), moderate (n = 1) or severe (n = 3) swelling of the left scrotum accompanied by hyperaemia of the scrotal skin. Three of these boars also presented with a mild swelling of the right scrota. There were no obvious signs of pain or discomfort due to the swollen scrota in any of the boars. One Piétrain boar exhibiting scrotal swelling additionally suffered from grade II lameness on both hind limbs and mild swelling of the tarsal joints. The willingness to mount the phantom was present in all boars, except in the lame Piétrain boar. All other parameters of the clinical examination were in accordance with the physiological norm (for detailed information see Additional file 1).

### Ultrasonographic examination

**Fig. 2:**
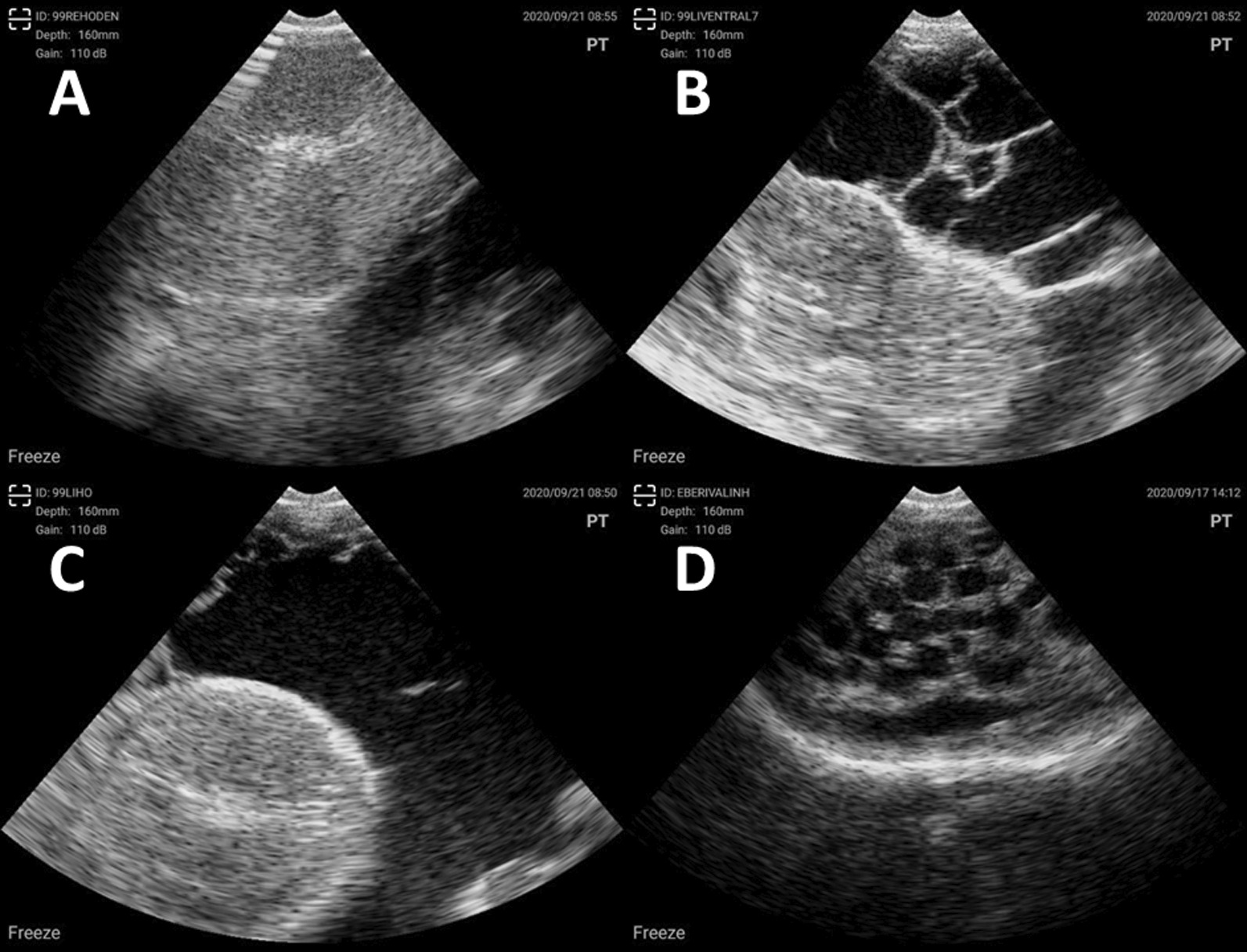
**A** Representative transversal ultrasound image of the non-affected testis with homogenous parenchyma and hyperechoic mediastinum from a case of unilateral scrotal swelling. Irregular hypoechoic areas in the lower right corner refer to the opposite site of the swollen scrotum. **B** and **C** Representative transversal ultrasound images of the affected testis with slightly inhomogenous parenchyma, thickened-hyperechoic testicular cover and surrounded by multiple, irregular hypoechoic areas with echogenic particles. **D** Representative ultrasound image of the cauda epididymis corresponding to the affected testis with dilated ductus epididymis

During ultrasonographic (US) examination of the affected boars (Smart Scan (B), Wireless Vet Ultrasound, mechanical sector scan, 3.5 MHz, MS Schippers, Bladel, The Netherlands), the non-affected testes showed a normal homogenous parenchyma with hyperechoic mediastinum and septula of the testis (Fig. 2A). Images of the affected testis demonstrated a non-homogeneous parenchyma, a thickened-hyperechoic testicular cover surrounded by multiple, and irregular hypoechoic areas with echogenic particles (Fig. 2B, C). The *cavum vaginale* was severely filled with fluid and separated by septa in caverns (Fig. 2B). The cauda epididymis corresponding to the affected testis presented with a dilated ductus epididymis (Fig. 2D).

### Pathologic and histopathologic examination

**Fig. 3:**
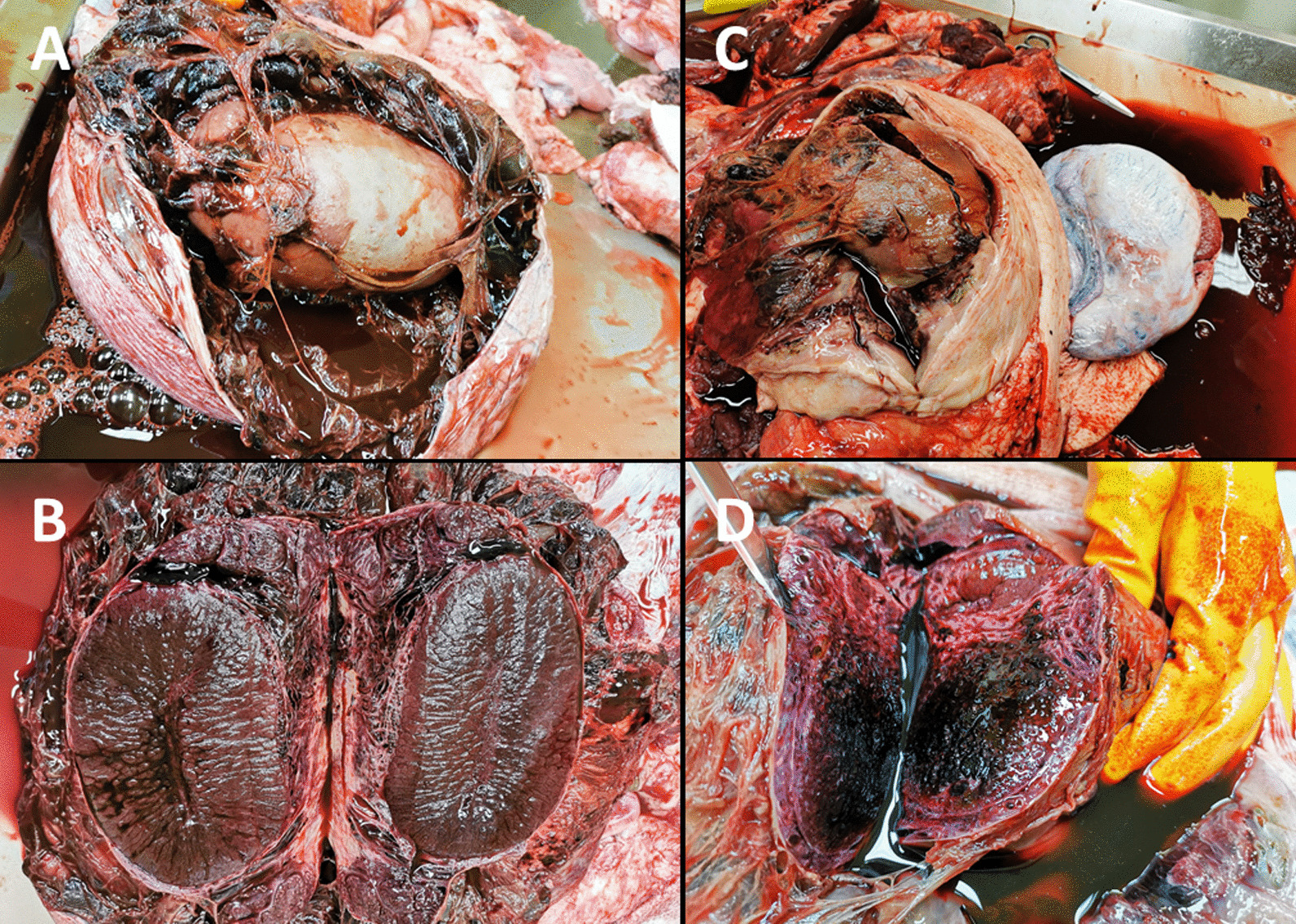
**A** High-grade swelling of the left scrotum with opened scrotal wall. Between the testicle and the scrotal wall there are multiple chambers, which were filled with large amounts of serous-bloody fluid and fibrinous tags or incipient adhesions; **B** Semi-circular incision through left testicle and epididymis. Necrosis of epididymal and testicular tissue with central acute haemorrhage; **C** High-grade enlargement of the left scrotum. The scrotal wall is open and shows a high degree of thickening due to connective tissue and oedema. The testicle is extensively adnate with the scrotal wall. In the gap between the testicle and the scrotal wall, there is a large amount of serous-bloody fluid and fibrinous tags or incipient adhesions; **D** Semi-circular incision through the testicle with high-grade, complete, acute haemorrhagic necrosis

For pathologic and histopathologic investigations, the five boars were anesthetized and euthanatized. Gross pathologic lesions were mainly restricted to the scrotum and testicles. Dissection of severely swollen scrota (n = 3) resulted in the release of massive amounts of blood-tinged fluid (Fig. 3A). In addition, fibrinous tags or incipient adhesions were detected between the testicles and scrotum (Fig. 3A and C; Additional file 1). Affected testicles had central acute haemorrhage (Fig. 3B and D). The penis and the accessory sex glands of all necropsied boars (n = 5) did not show any gross abnormalities. No puncture wounds were observed or detected, neither in the scrotal skin nor in the testicles.

**Fig. 4:**
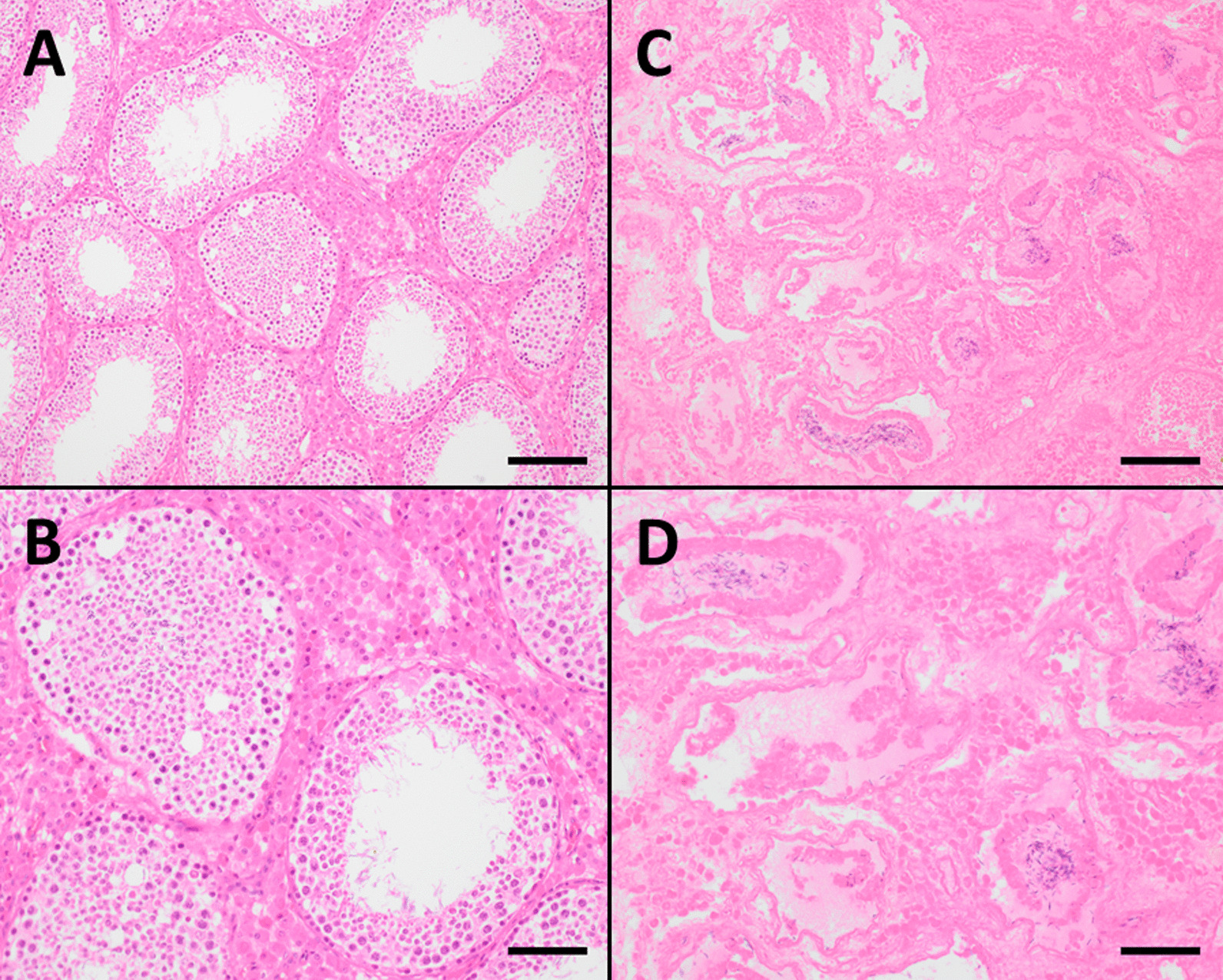
**A** and **B** Testicular tubules with mature spermatozoa in the lumen, which are partially filled by supporting cells and by cells of spermiogenesis. Leydig cells in the interstitial tissue; **C** and **D** Complete necrosis of the entire testicular tissue including the interstitium. Mature spermatozoa are still visible in the centre of some testicular tubules. Scale bar **A** and **C** 160 µm; **B** and **D** 80 µm

Histopathologic examination revealed complete acute haemorrhagic necrosis of the left testis in three boars (Fig. 4C and D), whereby the left epididymis also showed a complete acute tissue destruction with severe acute haemorrhages. The wall of the scrotum was highly thickened due to an increased formation of connective tissue with partly dystrophic calcifications; this was accompanied by an acute purulent inflammatory reaction as well as a significant oedema and blood congestion. In one boar, only a left-sided oedema of the epididymis and spermatic cord as well as a hydrocele could be detected. The remaining urogenital tract (urinary bladder, kidneys, accessory sex glands, penis) of the four aforementioned boars did not show any abnormal histopathology. One boar did not have lesions on the urogenital tract or other organ systems.

### Microbiologic investigations

Sera from the boars were tested for the presence of anti-*Brucella* spp. antibodies using Rose-Bengal test and complement fixation test. Additionally, different organ samples (testes, epididymides and accessory sex glands) were investigated for the presence of cultivable stages of *Brucella* spp. All investigations for direct or indirect detection of *Brucella* were negative. Scrotal aspirates, testicular tissue, epididymal tissue, tissue of accessory sex glands (bulbourethral, prostate and vesicular gland) and swabs of the urinary bladder were submitted for bacteriological investigation. In addition to bacteriological cultivation, PCRs of testicular tissue, scrotal aspirate, epididymal tissue and accessory sex gland tissue for *Brucella* spp., *Leptospira* spp., *Chlamydia* spp., *Glaesserella parasuis* and *Mycoplasma hyorhinis* were performed. None of the five bacterial pathogens could be detected in any of the animals. Cultivation of testicular and epididymal tissue revealed growth of sporadic/low grade *E. coli*, low grade *Staphylococcus chromogenes*, sporadic *Mammaliicoccus sciuri*, low grade *Streptococcus suis* and *Streptococcus alactolyticus* (overview on individual results see Additional file 1). The swabs of the urinary bladder also showed low levels of *E. coli* in three of five boars. However, urine of those boars did not exhibit any bacterial growth. The cultivation of the scrotal aspirate resulted in no detection of cultivable bacteria.

In addition, serological investigations of the examined boars and two additional animals for *Leptospira* spp. and *Chlamydia* spp. was performed (Additional file 1), with no remarkable findings.

### Virological investigations

All five necropsied boars were investigated for the presence of specific nucleic acids for African swine fever virus, pestiviruses (classical swine fever virus, atypical porcine pestivirus), PRRSV, porcine herpesviruses (including suid herpes virus 1) and flaviviruses (including Japanese encephalitis virus) using PCRs. In addition to the PCR-based investigations, attempts to isolate cytopathogenic viruses in an immortalized swine testicle cell line (ST-cells) were performed using serum and tissue of testicles and epididymides. All virological investigations resulted in negative results.

### Inspection of boar stud and further investigations

As the differential list of infectious agents possibly being involved in the aetiology of the swollen scrota and testes was ruled out, the investigative team focused on non-infectious causes during our on-site facility inspection. To find out possible causes for technopathic injuries and traumata, the whole boar stud was inspected for corresponding sources (for example, pen construction, facility design, barn equipment, doors, phantom, and semen collection rooms). Employees of the boar stud and the herd veterinarian described that nothing was changed regarding the daily routine procedures (for example, feeding, semen collection, cleaning of barns, handling of boars, etc.) over the past five years. The investigation team could not find any points, neither technically nor procedurally, which would be a plausible source for traumata resulting in testicular swellings. During the team’s presence, handling of boars by animal caretakers and animal technicians during guidance from the pen to the semen collection centre and back were done very gently without any use of handheld technical devices.

A commercial dry feed was used at the stud that was of consistent feed formulation and was fed to all boars. Given seasonal and regional differences in feed components, it was the only factor that changed over time from batch to batch. Mycotoxins may have been involved in the aetiology of the case. Within mycotoxins, especially zearalenone (ZEN) is known to cause fertility issues in livestock including atrophy of boar´s testes [7]. Therefore, a feed sample was analysed for mycotoxins and related metabolites in the framework of DSM mycotoxin survey program (Spectrum 380®) in the Centre for Analytical Chemistry, University of Natural Resources and Life Sciences, Vienna [8]. Results showed that mycotoxin concentrations were below the European guidance level or maximum content for complete feed (Additional file 2) [9–11].

Evaluation of sperm morphology was done on ejaculates of four out of five necropsied boars, with abnormal sperm morphology ranging from 34.5% to 92% (Additional file 3). Semen was collected by the herd veterinarian at the boar stud prior to movement of boars to the University Clinic for Swine. It was impossible to collect semen in the case of the fifth boar (with lameness), therefore no semen analysis was conducted. As it was already known from semen analysis done by the responsible herd veterinarian that semen quality of all affected boars was not meeting standards to be used in a breeding program, it was decided not to perform a more complete spermiogram due to financial reasons.

### Recommendation to the herd veterinarian

All investigations regarding infectious and non-infectious causes for swollen scrota resulted in negative outcomes leading to inconclusive results of the investigations. In order to prospectively obtain data in the case of clinical sign reoccurrence, the recommendation for the responsible herd veterinarian was to install video recording cameras for the continuous observation and evaluation of the boars’ health, after previously asking for a written consent of all employees to fulfil legislative requirements of the Austrian data protection regulation. It was recommended to install cameras at least in the semen collection area and in the corridor of each row of boar pens.

## Discussion and conclusion

**Fig. 5:**
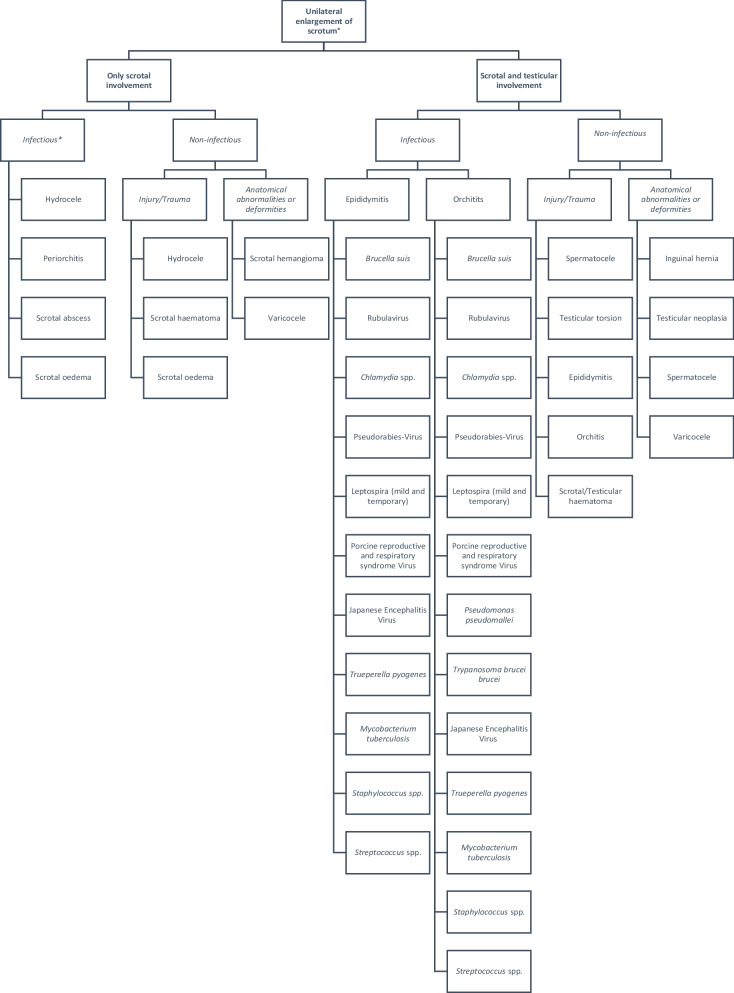
An overview and summary of differential diagnoses in case of unilateral enlargement of the scrotum. This decision tree is the base for similar cases in future to make the diagnostic workup of similar cases more systematic and complete. ° A proper clinical examination and knowledge is needed to get not misled from a physiologically sized testis and testis with a reduced size caused by different reasons. * Most frequent causes for scrotal involvement are due to infectious agents of the testicles. However, infection due to ubiquitous pathogenic agents only including the scrotum are possible

This case report describes an episodic occurrence of unilateral/bilateral scrotal swelling in boars of an Austrian boar stud. To the authors’ knowledge, no scientific literature describes the clinical and the pathologic presentation of a group of adult boars exhibiting unilateral and bilateral scrotal swelling. However, single cases have been described [12, 13]. The purpose of this case report was to summarize the findings and current evident differential diagnoses to offer a scientific base for similar cases in future (Fig. 5). It rather should point out the necessity of collaboration in science locally and abroad to proceed in gaining knowledge, especially in the case of boar management. The decision tree in Fig. 5 should give an overview of possible differential diagnoses and may help in similar cases in future to clarify the aetiology.

Scrotal swelling in boars may have different etiologic reasons; it can develop due to infections, but may also occur after traumatic injury [14–17]. Creating a list of differential diagnoses which may explain such clinical signs is essential but might be difficult especially in the case of adult boars, as the scientific literature is limited [18, 19]. Therefore, personal experience shared by veterinarians working in boar studs is very valuable in helping to create a robust list of differential diagnoses, especially when the issue has barely been tackled in the scientific literature. This case report supports this latter statement, as only the connection to other researchers and experienced people in the field of boars/boar studs and/or theriogenology led to a complete differential diagnosis list where differentials were eliminated, leaving a final presumptive diagnosis of a blunt force trauma to the affected boars.

During the diagnostic workup, it is necessary to carefully evaluate each result and to discuss its plausibility and validity. Starting with one of the most plausible differential diagnosis—porcine brucellosis—each further differential diagnosis should be excluded step by step. A long list of differentials leads to high costs, therefore a stepwise diagnostic workup may help keeping costs lower. Rubula virus, which is known to cause testicular swelling was not investigated, because this viral disease is only described to occur in central Mexico and has never been reported elsewhere [20]. In the diagnostic workup of this case, we could not definitively identify any infectious or non-infectious agent known to cause scrotal or testicular swelling in boars, despite the fact that acute inflammatory reactions were observed via histopathology of the scrotal wall. This finding is believed to be the consequence of chemo-attraction of neutrophils by necrotic material [21]. Moreover, we even included infectious agents in the list of differential diagnoses, such as *Glaesserella parasuis* and *Mycoplasma hyorhinis*, which have not been definitively ascribed to scrotal swelling outcomes but, through their pathogenesis, may be possible. This was due to the observed caverns filled with blood-tinged fluid in testicles/scrota during necropsy, as we could not exclude main polyserositis-causing agents [22] which may have led to fluid and fibrin accumulation in the *cavum vaginale* of testicles/scrota.

In case of *Leptospira* microscopic agglutination testing (MAT), two boars showed titres against some serovars at one time point. As boars were euthanized and no previously sampled sera were available at the time when boars were investigated at the University of Veterinary Medicine Vienna, we were not able to conclude on the relevance of the measured *Leptospira* antibody titres (1:100–1:200), as MAT is a method primarily developed for herd investigations or for investigating acute infections in single animals in case acute and convalescent samples show a four-fold rise [23]. It is unlikely that *Leptospira* spp. were involved in this clinical case, as only two out of five investigated boars showed any antibody titres. Regardless, based on the non-negative *Leptospira* results, the herd veterinarian decided to vaccinate boars at this stud against leptospirosis in the future.

During the inspection of the boar stud, sources of potential technopathic injuries were evaluated, but since the daily routine procedure and the housing system did not change over at least five years and no obvious sources of technopathic injuries were observed by us at our visit, we can only speculate about the definitive cause of the swollen scrota. Animal induced trauma, such as boars scaling the solid pen wall followed by an accidental fall on the testes due to a slippery floor could be one explanation for the swollen scrota. However, neither the floor and/or bedding material were slippery nor was scaling of the solid pen walls by boars observed by any workers in the boar stud. During our visit with the boar stud employees responsible for taking care of the boars, we found their interaction with the boars to be very pleasant and gentle. This case occurred in 2020 when the global coronavirus disease 2019 (COVID-19) pandemic started. In Austria, as in many other countries all over the world, lockdowns were ordered by the government to reduce virus transmission through social distancing. Travelling also became complicated compared to pre-pandemic times, as in the early phases of the first lockdown in Austria, expensive PCR tests were needed to travel from one country to another. Workers in the boar stud were of multiple ethnicities and have been working there for several years. Workers of the boar stud reported that they were concerned and decided not to visit their homes in eastern European countries. This change in the travelling behaviour due to the pandemic situation led to psychological distress in many people as recently reported [24, 25]. Mental impairment, such as depression and anxiety [26], combined with restrictions to public life and/or independence ordered by the government may have contributed to aggressive and frustrated behaviours in boar stud workers. The responsible herd veterinarian, who was also the owner of the boar stud, reported that farm workers were frustrated at least to a certain degree, as they were isolated from their families and were not able to visit their homes.

After eliminating other differentials, the pathological lesions observed in this case most likely were caused by a traumatic event that led to a local hematoma and consecutively to vascularization disorders finally resulting in massive fluid accumulation in the *cavum vaginale*. As the herd veterinarian reported that no animal-induced trauma events (for example, falling off the dummy or scaling pen walls) happened during semen collection and no sources of technopathies could be detected, we speculate that human mistreatment against boars may have led to blunt traumata. Six out of eight boars were kept in pens of one row which had to be re-entered making a left turn. Before final entry into the pen the left scrotum/testicle was exposed. In case the boars may have stolidly re-entered, one may speculate that workers may have inflicted a physical event to the rear of the boar using an object or body part (for example, feet) to accelerate the re-entry of the boar. Although we know that the latter statement is speculative, it cannot be excluded and should be kept in mind.

In case of suspected animal abuse validated, repeatable and feasible animal-based measures have to be implemented in a farm to guarantee a high level of animal welfare and to create awareness of animal welfare in all persons involved in handling of animals [27]. The responsible herd veterinarian was recommended to install video recording cameras for the observation and evaluation of boars’ health. He only started the discussion process of camera installation for health observation of boars together with the employees, but decided not to do so, as the workers were quite irritated and concerned about being observed. Indeed, such discussions need a high grade of tactfulness and discretion as it may be evaluated by workers as lack of confidence. However, after having discussions with the workers regarding the process of camera installation, no further incidents of unilateral/bilateral swollen scrota have occurred at this stud to date.

Due to the fact, that we started to confirm or exclude brucellosis as the first suspected diagnosis, we may have been misled by infectious causes in our diagnostic work-up. For the future, practitioners may follow a proper clinical examination including available medical data existing in literature about imaging methods such as ultrasonography. This might have brought more insights into traumatic events as a possible cause of scrotal swelling prior to necropsy of boars [28–30]. Including different imaging techniques may have resulted in blunt trauma as suspected diagnose earlier, which might have enabled us to also investigate blood for break down products of haemoglobin and for iron storage capacity.

Although our diagnosis was through both gross and histopathologic findings along with differential list exclusion of known causes of unilateral swollen scrota and necrosis of testicles, we think that this case report is very important for future similar cases. It will serve both as a guideline for differential diagnoses in case of scrotal swelling and as a base for discussion of the role of possible human misbehaviour in boar studs.

## Supplementary Information

**Additional file 1.** Summary of all clinical and laboratory findings of the investigated boars.

**Additional file 2.** Summary of the mycotoxicologic investigations of feed.

**Additional file 3.** Results of semen analysis of collected semen.

## Data Availability

All data generated or analysed during this case report are included in this published article or are available as supplementary data.

## Abbreviations

MAT: Microscopic agglutination test
PCR: Polymerase chain reaction
PRRSV: Porcine reproductive and respiratory syndrome virus
SARS-CoV-2: South Asian respiratory syndrome coronavirus 2
ST-cells: Swine testicle cells
ZEN: Zearalenone
