# Supplementary material for: A single, episodic event of unilateral/bilateral scrotal swelling in a group of adult boars at an Austrian boar stud
Source: Porcine Health Manag. 2023 Jul 14;9:17. doi: 10.1186/s40813-023-00313-1 (PMC10347806; doi:10.1186/s40813-023-00313-1)
Supplement: Supplementary file 2 — Additional file 2. Summary of the mycotoxicologic investigations of feed. [file 40813_2023_313_MOESM2_ESM.docx]

Table 1: Main mycotoxins identified with Spectrum 380® - method

| **Toxin** | **Numbers of toxin within this group** | **Concentration**  **(ppb = µg/kg)** | **Guidance level/maximum content of the European Commission for complete feed**  **(ppb = µg/kg)** | **Legislative basis in the EU** |
| --- | --- | --- | --- | --- |
| A-Trichothecene^1^ | 1 | 3.88 | 250^6^ | 2013/165/EU |
| B-Trichothecene^2^ | 1 | 128.4 | * | 2006/576/EU |
| Ergotalkaloide^3^ | 3 | 1.88 | ** | 2002/32/EC |
| Fumonisine^4^ | 4 | 157.93 | 5,000^7^ | 2006/576/EU |
| Zearalenone-metabolite^5^ | 2 | 15.54 | 250^8^ | 2006/576/EU |
| Aflatoxin | - | < LOD | 20^9^ | 2002/32/EC |
| Ochratoxin A | - | < LOD | 50 | 2006/576/EU |

^1^T-2 toxin

^2^15-Acetyldeoxynivalenol

^3^Ergometrinine, Ergosin, Ergosinin;

^4^Fumonisin B1, B2, B3, B4

^5^Zearalenone, Zearalenone-Sulfate (no standard for quantification of Zearalenone-Sulfate available)

^6^ Guidance level just valid for T2 and HT2

^7^ Guidance level just valid for Fumonisine B1 and B2

^8^ Guidance level just valid for Zearalenone

^9^ Aflatoxin B_1_ maximum content for complete feed.

*Currently, only guidance levels for deoxynivalenol (DON) are established (900 ppb in compound feeds for swine), 15-Acetyldeoxynivalenol is completely hydrolyzed to DON pre-systemically

**Content of Ergotalkaloids is currently not regulated in European Union, but maximum content of Rye Ergot Sclerotia (1000 ppm).

Table 2: Other mycotoxins and metabolites identified with Spectrum 380® - method

| **Toxin** | **Numbers of toxin within this group** | **Concentration**  **(ppb = µg/kg)** | **Guidance levels** |
| --- | --- | --- | --- |
| Alternariatoxins | 7 | 598.18 | Not regulated  Not regulated  Not regulated  Not regulated  Not regulated  Not regulated |
| Aspergillustoxins | 2 | 11.21 |  |
| Enniatins and Beauvericin | 6 | 35.69 |  |
| Fusarium metabolites | 11 | 1550.63 |  |
| Penicillium toxins | 2 | 5.05 |  |
| Other metabolites | 1 | 250.01 |  |
